# Diabetes duration and health-related quality of life in individuals with onset of diabetes in the age group 15—34 years – a Swedish population-based study using EQ-5D

**DOI:** 10.1186/1471-2458-13-377

**Published:** 2013-04-22

**Authors:** Vibeke Sparring, Lennarth Nyström, Rolf Wahlström, Pia Maria Jonsson, Jan Östman, Kristina Burström

**Affiliations:** 1 Medical Management Centre, Department of Learning, Informatics, Management and Ethics, Karolinska Institutet, Tomtebodavägen 18 A, SE-17177 Stockholm, Sweden; 2 Epidemiology and Global Health, Department of Public Health and Clinical Medicine, Umeå University, SE-90185 Umeå, Sweden; 3 Division of Global Health, Department of Public Health Sciences, Karolinska Institutet, Tomtebodavägen 18 A, SE-17177 Stockholm, Sweden; 4 Family Medicine and Preventive Medicine, Department of Public Health and Caring Sciences, Uppsala University, P.O. Box 564 Uppsala, SE-75122, Sweden; 5 National Institute of Health and Welfare, P.O. Box 30, FI-00271 Helsinki, Finland; 6 Department of Medicine, Karolinska University Hospital, SE-14186 Huddinge, Sweden; 7 Division of Social Medicine, Department of Public Health Sciences, Karolinska Institutet, Tomtebodavägen 18 A, SE-17177 Stockholm, Sweden; 8 Health Care Services, Stockholm County Council, Tomtebodavägen 18 A, SE-17177 Stockholm, Sweden

**Keywords:** Diabetes mellitus, Disease duration, EQ-5D, Health-related quality of life, Sweden

## Abstract

**Background:**

Diabetes with onset in younger ages affects both length of life and health status due to debilitating and life-threatening long-term complications. In addition, episodes and fear of hypoglycaemia and of long-term consequences may have a substantial impact on health status. This study aims to describe and analyse health-related quality of life (HRQoL) in individuals with onset of diabetes at the age of 15—34 years and with a disease duration of 1, 8, 15 and 24 years compared with control individuals matched for age, sex and county of residence.

**Methods:**

Cross-sectional study of 839 individuals with diabetes and 1564 control individuals. Data on socioeconomic status and HRQoL using EQ-5D were collected by a postal questionnaire. Insulin treatment was self-reported by 94% of the patients, the majority most likely being type 1.

**Results:**

Individuals with diabetes reported lower HRQoL, with a significantly lower mean EQ VAS score in all cohorts of disease duration compared with control individuals for both men and women, and with a significantly lower EQ-5D_index_ for women, but not for men, 15 years (0.76, p = 0.022) and 24 years (0.77, p = 0.016) after diagnosis compared with corresponding control individuals. Newly diagnosed individuals with diabetes reported significantly more problems compared with the control individuals in the dimension usual activities (women: 13.2% vs. 4.0%, p = 0.048; men: 11.4% vs. 4.1%, p = 0.033). In the other dimensions, differences between individuals with diabetes and control individuals were found 15 and 24 years after diagnosis: for women in the dimensions mobility, self-care, usual activities and pain/discomfort and for men in the dimension mobility. Multivariable regression analysis showed that diabetes duration, being a woman, having a lower education and not being married or cohabiting had a negative impact on HRQoL.

**Conclusions:**

Our study confirms the negative impact of diabetes on HRQoL and that the difference to control individuals increased by disease duration for women with diabetes. The small difference one year after diagnosis could imply a good management of diabetes care and a relatively quick adaptation. Our results also indicate that gender differences still exist in Sweden, despite modern diabetes treatment and management in Sweden.

## Background

Diabetes with onset in younger ages affects both length of life and health status due to debilitating and life-threatening long-term complications [1]. In addition, episodes and fear of hypoglycaemia and of long-term consequences may have a substantial impact on health status [2,3]. Health-related quality of life (HRQoL) is a subjective assessment of health status that includes relevant aspects such as general health, physical, emotional, cognitive, and role functioning, as well as social well-being and functioning [4]. HRQoL for individuals with diabetes can be measured either by using a diabetes-specific instrument which may detect subtle disease and treatment-related effects [5-8], or generic instruments, which enables comparisons to the general population or other diseases [8-11]. Deciding on what type of instrument to use depends on what decisions you would like to make based on the results [12]. In order to compare both the difference between individuals with varying diabetes duration, and the difference between individuals with diabetes and the general population, a generic instrument is the most appropriate choice. The literature supports the use of generic HRQoL instruments for measuring health status in individuals with diabetes, e.g., Short Form 36 (SF-36), Health Utility Index (HUI), or EQ-5D [5,13,14]. The latter is a short instrument, which can be included in any questionnaire, and it has been used in the general population as well as on a wide range of health conditions and treatments [15].

Several studies using EQ-5D have found that individuals with diabetes rate their HRQoL lower than the general population [10,14,16-20]. The majority of these studies focused on individuals with type 2 diabetes or a mix of individuals with type 1 or type 2 diabetes, whereas there is a lack of studies focusing on individuals with type 1 diabetes. HRQoL is likely to be affected not only by having diabetes but also by disease duration. Intensive treatment in the early phases of the disease as well as current routine treatment have been shown to reduce or postpone the occurrence of long-term complications [21,22], which could reduce the negative impact of the disease on HRQoL. The increased focus on the disease and the balancing of all routines that follow to reach a stable glucose control may feel like a hindrance in life and may also lead to fear of hypoglycaemic reactions [3], which could have an impact on HRQoL. As treatment becomes routine, the deterioration in HRQoL can be expected to be reduced. However, as the disease progresses complications are likely to set in and result in a gradual decrease over time in HRQoL among individuals with diabetes beyond that of the general population. Several studies have shown that long-term complications of diabetes such as ischemic heart disease, stroke, neuropathy and retinopathy have a negative impact on HRQoL [9,23-27].

Few studies have used EQ-5D and compared results on individuals with varying duration of diabetes. It was found, in a Dutch study on 234 individuals with type 1 diabetes with an average disease duration of 17 years at entry, that self-rated health and functioning over time decreased faster for patients compared with the general population and that HRQoL decreased significantly per year of follow-up [14]. Patients living with a partner reported higher HRQoL, and, unexpectedly, higher educational level was associated with lower HRQoL [20]. HRQoL for married patients decreased slower over time compared with unmarried patients [28].

With this in mind, we hypothesised that sex, level of education, marital status as well as onset and disease duration would have an impact on HRQoL for individuals with diabetes. We used the EQ-5D instrument to facilitate comparison both with the control individuals in our study and with other studies of individuals with diabetes or other chronic diseases and the general population where EQ-5D has been used. This study thus aims to describe and analyse HRQoL in individuals with onset of diabetes at the age of 15—34 years and with a disease duration of 1, 8, 15 and 24 years, and to compare with control individuals matched for age, sex and county of residence.

## Methods

Participants were selected among all registered individuals in four incidence cohorts (1983, 1992, 1999 and 2008) in the Diabetes Incidence Study in Sweden (DISS). Since 1983, DISS prospectively registers incident cases of diabetes mellitus in Sweden in the age group 15 to 34 years, on average about 400 cases per year. Basic characteristics of patients at diagnosis are reported by the diagnosing doctor on a standardised form and the level of ascertainment is estimated at 80–90%. The majority of the patients in DISS (74%) are clinically classified as type 1 diabetes, 17% as type 2 and 9% as unclassified [29]. All individuals in the respective cohorts, regardless of type of diabetes, were included in this study. The choice of these four cohorts was based on time since diagnosis, connected to our hypotheses that different disease durations may have dissimilar effects on HRQoL.

For each individual with diabetes, two control individuals were randomly selected from the general population register in which demographic characteristics on Sweden’s population are reported by age, sex, civil status, citizenship, country of birth, migration, births and deaths [30]. The control individuals were matched by age, sex and county of residence at the time when the questionnaire was sent out. Age was matched by choosing the two individuals with the personal identification number (based on year, month and date of birth) closest to the individual with diabetes, usually born on the same day or the day before or after. Since the cohorts are selected based on year of diabetes diagnosis, the mean age in the different groups will differ, i.e. adding 1, 8, 15 and 24 years respectively to the mean of 15–34 years in each cohort. The HRQoL was measured by a postal questionnaire sent to all individuals. To increase the response rate a total of three reminders were sent to those who had not yet responded.

### Survey questionnaire

The present questionnaire addressed healthcare utilisation, use of drugs, questions on socioeconomic status, and HRQoL. It was based on a questionnaire applied in a previous follow-up study of the 1983 and 1992 cohorts [31], to which the EQ-5D instrument was added. For this study, we have analysed the EQ-5D questions and questions on socioeconomic status.

### The EQ-5D instrument

The EQ-5D is a standardised instrument used to measure health outcome both on a wide range of health conditions and treatments as well as in the general population [10,15]. With the EQ-5D measure respondents can classify their health status in five dimensions (mobility; self-care; usual activities; pain/discomfort; anxiety/depression) in three levels of severity (no, moderate or severe problems). By this classification, there are 243 possible unique health states that can also be converted into a single index value (EQ-5D_index_) for health status (1 = full health; 0 = dead). The index value was assigned by adopting the most commonly used value set, the York MVH A1 value set, which derives from valuations of health states representing the average preference of the general UK population [32]. The overall self-rated health score, EQ VAS score, was recorded on a scale from 0 (worst imaginable health state) to 100 (best imaginable health state).

### Data collection

The questionnaire was sent to the 1999, 1992 and 1983 year cohorts in January 2008, i.e., 8, 15 and 24 years after diagnosis, and was answered by 721 individuals with diabetes (response rate 58%) and 1360 corresponding control individuals (response rate 55%). To the 2008 year cohort and corresponding control individuals, the questionnaire was sent in 2009, i.e. 1 year after diagnosis, and to get as close as possible to the date of diagnosis of each individual with diabetes the questionnaire was sent quarterly (response rate 42%; n = 143 and 38%; n = 256 respectively). In the analyses, some individuals were excluded due to non-sampled persons having answered the questionnaire (n = 7), missing information on sex (n = 2), missing information on age (n = 3), or having incomplete or ambiguous answers on the EQ-5D dimensions (n = 66; 2.6%). After exclusion, the sample consisted of 839 patients and 1564 control individuals. The proportion of individuals with self-reported insulin treatment at follow-up was 94%. The proportions of men, individuals below 30 years of age and not being married or cohabiting were significantly higher among the non-responders but did not differ between individuals with diabetes and control individuals.

### Statistical analyses

Data were analysed using SPSS. The main outcome measures were self-reported health as expressed in the five EQ-5D dimensions, the EQ-5D_index_ and the EQ VAS score. To test whether there was a difference in sociodemographic characteristics between patients and control individuals in the different cohorts, and the percentage of reported problems in the EQ-5D dimensions, Pearson’s χ^2^-test or Fisher’s exact test was used. The categories moderate and severe problems were collapsed before testing. Independent samples t-test was used to test whether there were differences in the mean EQ-5D_index_ and EQ VAS score between diabetes individuals with different disease duration. Comparisons between individuals with different diabetes duration were carried out using one-way ANOVA. Post-hoc tests were performed with Bonferroni and Dunnett T3 tests. The same tests were applied for the control individuals.

Multivariable regression analysis was performed to identify the independent variables (sex, diabetes diagnosis, disease duration, level of education, and marital status) that could predict the variation in the dependent variables EQ-5D_index_ and EQ VAS score. Level of education was dichotomised into primary school or lower and secondary school or higher and marital status was dichotomised into married or cohabiting and not married or cohabiting. All statistical tests were carried out at 5% significance level.

### Ethical approval

Informed consent was obtained through an accompanying letter to the questionnaire explaining the study. All analyses were carried out on group level and traceability to individuals is therefore not possible. The study was approved by the Regional Ethics Committee in Stockholm (2007/214-31).

## Results

### Characteristics of the sample

There were no differences between patients and control individuals regarding educational level and marital status except that individuals with 1-year diabetes duration had lower educational level than their control individuals and that the proportion of married or cohabiting was lower among individuals with diabetes 1 and 8 years after diagnosis than among control individuals (Table 1). When stratifying for sex, the above mentioned significances were all attributed to men with diabetes.

**Table 1:** Percentage of distribution of socio-demographic characteristics of individuals with diabetes and control individuals by disease duration

	**Disease duration**							
	**1 year**		**8 years**		**15 years**		**24 years**	
	**(2008 cohort)**		**(1999 cohort)**		**(1992 cohort)**		**(1983 cohort)**	
	**Individuals with diabetes**	**Control individuals**	**Individuals with diabetes**	**Control individuals**	**Individuals with diabetes**	**Control individuals**	**Individuals with diabetes**	**Control individuals**
	**n = 141**	**n = 247**	**n = 208**	**n = 381**	**n = 225**	**n = 419**	**n = 265**	**n = 517**
	**%**	**%**	**%**	**%**	**%**	**%**	**%**	**%**
**Age (year)**^†^								
16-24	45.4	44.1	6.7	6.0	-	-	-	-
25-34	45.4	47.0	40.4	39.9	20.0	22.2	-	-
35-44	9.2	8.9	52.9	54.1	50.2	48.0	18.1	18.3
45-54	-	-	-	-	29.8	29.8	50.6	48.5
55+	-	-	-	-	-	-	31.3	33.2
**Age (years, mean)**								
Women	25.6	26.9	34.5	34.4	40.7	40.2	49.8	50.2
Men	26.1	25.5	33.7	34.2	40.2	40.1	49.7	49.7
**Sex**								
Women	37.6	40.9	45.2	44.1	41.8	41.1	42.3	39.1
**Level of education**								
Primary school or lower	71.0	56.3*	55.8	50.5	65.9	59.6	67.6	61.9
Secondary school or higher	29.0	43.7	44.2	49.5	34.1	40.4	32.4	38.1
**Marital status**								
Married or cohabiting	40.3	51.0*	70.2	78.7*	74.7	75.2	74.3	75.6
Not married or cohabiting	59.7	49.0	29.8	21.3	25.3	24.8	25.7	24.4

^†^Age at the end of the year when the respondents received the questionnaire, i.e. 2009 for the 2008 cohort followed-up 1 year after diagnosis and 2007 for the 1983, 1992 and 1999 cohorts followed-up 8, 15 and 24 years after diagnosis.

^*^ Significant differences were found in comparisons between patients with diabetes and control individuals with 95% significance level.

### Reported problems in EQ-5D dimensions

Compared with control individuals, both women and men with diabetes reported significantly more problems in the dimension usual activities 1 year after diagnosis (Table 2). Eight years after diagnosis, women with diabetes reported more problems in the dimension pain/discomfort than the control individuals. Fifteen years after diagnosis, women with diabetes reported more problems in the dimensions usual activities and pain/discomfort than their corresponding control individuals, and men with diabetes reported more problems in the dimension mobility than their corresponding control individuals. In the dimension pain/discomfort, men with diabetes reported less problems than the corresponding control individuals. Twenty-four years after diagnosis, women reported more problems in the dimensions mobility, self-care and usual activities than female control individuals.

**Table 2:** Percentage of patients with diabetes and control individuals reporting moderate and severe problems in each EQ-5D dimension by disease duration

	**Diabetes duration**																					
	**1 year**					**8 years**					**15 years**					**24 years**					**Comparison of individuals with diabetes**	**Comparison of control individuals**
	**(2008 cohort)**					**(1999 cohort)**					**(1992 cohort)**					**(1983 cohort)**						
	**Individuals with diabetes**		**Control individuals**		***p***^**a**^	**Individuals with diabetes**		**Control individuals**		***p***^**a**^	**Individuals with diabetes**		**Control individuals**		***p***^**a**^	**Individuals with diabetes**		**Control individuals**		***p***^**a**^		
	**n = 141**		**n = 247**			**n = 208**		**n = 381**			**n = 225**		**n = 419**			**n = 265**		**n = 517**			***p***^**b**^	***p***^**c**^
	**%**	**n**	**%**	**n**		**%**	**n**	**%**	**n**		**%**	**n**	**%**	**n**		**%**	**n**	**%**	**n**			
**Mobility**																						
Women																						
Moderate problems	5.7	3	4.0	4	0.69	6.4	6	3.6	6	0.36	11.7	11	9.3	16	0.54	16.1	18	9.9	19	0.049	0.053	0.053
Severe problems	0.0	0	0.0	0		0.0	0	0.0	0		0.0	0	0.0	0		0.9	1	0.0	0			
Men																						
Moderate problems	2.3	2	2.7	4	1.00	2.6	3	5.6	12	0.22	10.7	14	4.9	12	0.033	17.6	27	15.9	50	0.51	<0.001	<0.001
Severe problems	0.0	0	0.0	0		0.0	0	0.0	0		0.0	0	0.0	0		0.7	1	0.0	0			
**Self-care**																						
Women																						
Moderate problems	0.0	0	0.0	0	-	0.0	0	0.6	1	1.00	1.1	1	2.3	4	0.43	2.7	3	0.5	1	0.023	0.048	0.059
Severe problems	0.0	0	0.0	0		0.0	0	0.0	0		0.0	0	0.6	1		1.8	2	0.0	0			
Men																						
Moderate problems	1.1	1	0.0	0	0.38	1.8	2	0.9	2	0.61	3.8	5	1.6	4	0.10	3.3	5	1.9	6	0.58	0.37	0.11
Severe problems	0.0	0	0.0	0		0.0	0	0.0	0		0.8	1	0.0	0		0.7	1	1.0	3			
**Usual activities**																						
Women																						
Moderate problems	13.2	7	3.0	3	0.048	12.8	12	7.7	13	0.18	14.9	14	6.4	11	0.029	20.5	23	8.4	16	0.001	0.19	0.42
Severe problems	0.0	0	1.0	1		0.0	0	0.0	0		2.1	2	1.7	3		2.7	3	1.5	3			
Men																						
Moderate problems	9.1	8	3.4	5	0.033	4.4	5	6.6	14	0.64	9.9	13	7.7	19	0.31	12.4	19	8.6	27	0.14	0.090	0.065
Severe problems	2.3	2	0.7	1		1.8	2	0.9	2		2.1	3	1.2	3		3.9	6	2.9	9			
**Pain/discomfort**																						
Women																						
Moderate problems	28.3	15	25.7	26	0.35	45.7	43	31.5	53	0.050	48.9	46	35.5	61	0.013	51.8	58	47.5	96	0.33	0.041	<0.001
Severe problems	5.7	3	1.0	1		1.1	1	3.0	5		5.3	5	2.9	5		4.5	5	3.0	6			
Men																						
Moderate problems	22.7	20	24.7	36	0.77	28.9	33	32.4	69	0.41	24.4	32	40.1	99	0.012	49.7	76	44.8	141	0.12	<0.001	<0.001
Severe problems	2.3	2	2.1	3		0.9	1	1.9	4		6.1	8	3.6	9		7.2	11	4.4	14			
**Anxiety/depression**																						
Women																						
Moderate problems	32.0	17	30.7	31	0.60	36.2	34	27.4	46	0.13	38.3	36	29.7	51	0.057	46.4	52	36.0	73	0.056	0.29	0.41
Severe problems	3.8	2	1.0	1		2.1	2	1.8	3		8.5	8	5.2	9		1.8	2	1.0	2			
Men																						
Moderate problems	28.4	25	23.3	34	0.14	30.7	35	24.9	53	0.13	25.2	33	31.6	78	0.22	31.4	48	23.2	73	0.064	0.43	0.28
Severe problems	6.8	6	2.7	4		3.5	4	1.4	3		1.5	2	1.2	3		3.3	5	3.2	10			

^a^ P-value for comparison between patients with diabetes and control individuals (χ^2^ or Fisher exact test), ^b^ p-value for comparison between patients with diabetes in the different cohorts (ANOVA), ^c^ p-value for comparison between control individuals in the different cohorts (ANOVA).

In general, the combined prevalence of moderate and severe problems increased in all five dimensions by disease duration for individuals with diabetes. However, significant differences between individuals with a disease duration of 15 or 24 years compared to those with a shorter duration, could only be seen for self-care and pain/discomfort among women and for mobility and pain/discomfort among men. Similar overall patterns were found for control individuals with significantly more problems with pain/discomfort among women and with mobility and pain/discomfort among men.

### EQ-5D_index_ and EQ VAS score

**Figure 1:**
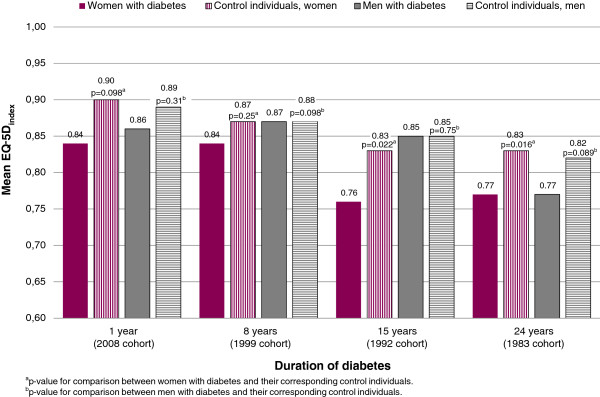
**Mean EQ-5D**_**index **_**in individuals with diabetes compared to control individuals by disease duration.** Comparison (ANOVA) of the cohorts for the different years of disease duration found significant differences between the different cohorts of women with diabetes (p = 0.022), of female control individuals (p = 0.011), of men with diabetes (p = 0.002) and of male control individuals (p = 0.001), respectively. Post hoc tests could not detect between which cohorts the significance for women with diabetes lay. The test, however, showed significant differences men with diabetes between the 1983 cohort and all other cohorts (1992, p = 0.002; 1999, p = 0.007; 2008, p = 0.027), for female control individuals between the 2008 and 1983 cohorts (p = 0.025), and for male control individuals between the 1983 cohort compared with the 2008 (p = 0.002) and 1999 (p = 0.009) cohorts.

Among women, mean EQ-5D_index_ was significantly lower for individuals with diabetes compared with control individuals 15 and 24 years after diagnosis (Figure 1). Comparison of women with diabetes in the different cohorts showed significant differences in mean EQ-5D_index_ between the different cohorts of women with diabetes, which also applied for the control individuals. A similar pattern was seen for men with diabetes as well as for male control individuals. Post hoc tests could not detect any significant differences between cohorts for women with diabetes, while significant differences were found for men with diabetes between the 1983 cohort and all other cohorts (Figure 1). For female control individuals, significant differences were found between the 1983 and 2008 cohorts, and for male control individuals between the 1983 cohort and the 2008 and 1999 cohorts (Figure 1).

**Figure 2:**
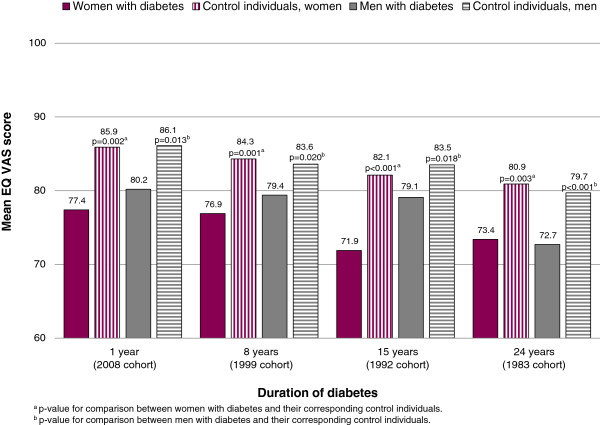
**Mean EQ VAS scores in individuals with diabetes compared to control individuals by disease duration.** ANOVA and post hoc tests showed significant differences between the cohorts of men with diabetes (p = 0.004) which were found between the 1983 cohort and the 2008 (p = 0.020) and 1999 (p = 0.028) cohorts. Between the cohorts of male control individuals significant differences were found (p < 0.001) between the 1983 cohort and all other cohorts (1992, p = 0.045; 1999, p = 0.040; 2008, p < 0.001). The post hoc tests could not detect any significant differences between the cohorts for women, neither for women with diabetes nor for the control individuals.

Mean EQ VAS scores were significantly lower for both women and men with diabetes compared with control individuals in all cohorts (Figure 2). Comparisons showed no differences between the cohorts of women with different diabetes duration, and the same applied for their corresponding control individuals. However, for men with diabetes significant differences were found between the different cohorts, which was also the case for their control individuals (Figure 2).

### Variation on health-related quality of life

The multivariable regression analysis showed similar patterns for both EQ-5D_index_ and EQ VAS score used as health outcomes measures (Table 3). The model showed that sex, being diagnosed with diabetes, level of education and not being married or cohabiting all had significant negative impact on the health outcome measures along with disease duration of 8 years for EQ VAS score and disease duration of 15 and 24 years for both EQ-5D_index_ and EQ VAS score. We also tested models adding the independent variables separately but that did not significantly impact the final model.

**Table 3:** **Multiple regression analyses on EQ-5D**_**index **_**and EQ VAS score**

***Dependent variable:***	***EQ-5D***_***index***_		***EQ VAS score***	
	***ß***	***p***	***ß***	***p***
*Intercept*	0.930	<0.001	89.4	<0.001
*Sex*				
Men	ref.		ref.	
Women	−0.024	0.005	−1.8	0.014
*Diabetes diagnosis*				
Control individuals	ref.		ref.	
Patients with diabetes	−0.025	0.007	−6.0	<0.001
*Diabetes duration*				
1 year	ref.		ref.	
8 years	−0.023	0.099	−2.8	0.015
15 years	−0.055	<0.001	−3.8	0.001
24 years	−0.074	<0.001	−6.0	<0.001
*Level of education*				
Secondary school or higher	ref.		ref.	
Primary school or lower	−0.095	<0.001	−7.5	<0.001
*Marital status*				
Married or cohabiting	ref.		ref.	
Not married or cohabiting	−0.046	<0.001	−4.8	<0.001
*R-Square*	0.058		0.084	
*Adjusted R-Square*	0.056		0.082	
*Number of observations*	2384		2329	

## Discussion

This study showed that self-reported mean EQ VAS score was significantly lower for individuals with diabetes for all cohorts of disease duration compared with matched control individuals. Mean EQ-5D_index_ was significantly lower for women, but not for men, 15 and 24 years after diabetes diagnosis compared with corresponding female control individuals. One year after diagnosis, both women and men with diabetes reported significantly more problems in the dimension usual activities compared with corresponding control individuals. In the other dimensions, differences were found 15 and 24 years after diagnosis when comparing individuals with diabetes and control individuals. For women the differences were found in the dimensions mobility, self-care, usual activities and pain/discomfort and for men in mobility.

To be diagnosed with diabetes is an upheaval in life that, for the newly diagnosed, affects the EQ-5D dimension usual activities as well as mean EQ VAS score. Our assumption was that HRQoL would be negatively affected when treatment is in an intensive phase and that the dimension anxiety/depression would be significantly affected, but this was not supported by our results. Furthermore, there was no significant difference at this stage of the disease in EQ-5D_index _between individuals with diabetes and corresponding control individuals. The fact that the patients in the 1-year cohort reported their problems a whole year after onset, can explain why there was no discernible difference in EQ-5D dimensions compared with 8 years after diagnosis except for the dimension usual activities. It is likely that the patients one year after diagnosis have accepted the disease and somewhat adapted the new routines into their daily life. It has been indicated previously that although HRQoL decreases at onset it improves already within the first year to levels comparable to the general population [33].

Of all EQ-5D dimensions, problems were most prominent in the dimension pain/discomfort, which has also been shown in previous studies [10,14,16-20]. As stated previously, no significant differences were found in the dimension anxiety/depression one year after diagnosis between individuals with diabetes and control individuals and this also applied 8, 15 and 24 years after diagnosis. This could possibly be explained by a high prevalence of problems with anxiety/depression among the general population and also in younger age groups [10].

A decrease in EQ-5D_index_ and EQ VAS score for individuals with diabetes could be seen for both women (after 15 years) and men (after 24 years), and is consistent with Hart et al. [14], who showed a decrease in HRQoL per year with diabetes. Although women generally tend to rate their HRQoL lower than men [10,17], it is noteworthy that the difference between individuals with diabetes and control individuals increased after 15 years for women and after 24 years for men suggesting an earlier social stratification in health for women with diabetes compared with women in the general population as well as compared with men with diabetes. Two of the cohorts in this study (1983 and 1992) were also followed-up in the early 1990’s [31]. Although a different instrument was used, our results are similar to those findings with sex and socioeconomic factors being closely associated with self-rated health 1 year after diagnosis, as well as 8 years after diagnosis where the association was even stronger.

Our study must be seen in relation to the development in the management of diabetes in Sweden, which has changed considerably during the last decades. These changes include multiple daily injection regimens, use of insulin pumps, new insulin analogues as well as an increased proportion of self-monitoring and self-management. Changes have also been made in the provision of health services such as a shift from hospital inpatient care to daycare and a shift from visits to physicians to visits to diabetes nurses [34], and in the involvement of patients as part of the expert team around the disease with self-management as an important component [35]. Thus, the patients in this study have received different types of care dependent on the year of diagnosis. These changes in diabetes management likely contribute to reductions or postponements in diabetes-related complications, complications which may have a negative effect on HRQoL for individuals with diabetes.

A potential limitation of the study is the relatively low response rate, especially in the 2008 cohort, which could be a reflection of generally declining response rates in population and patient surveys also experienced by Statistics Sweden [36]. The strength of the study is the large number of participants, their entry at time of diagnosis and the long disease duration. The differences in socio-demographic factors between responders and non-responders may suggest worse health and lower socioeconomic status among the non-responders, but this applies equally to individuals with and without diabetes, implying a non-differential misclassification bias. Including young people in study populations may be problematic for analyses on socioeconomic factors such as educational level as some of them are still students. There is also a potential non-differential misclassification regarding marital status for the population below 20 years of age as most of them are likely to still live with their parents. This could lead to an overestimation of the positive effect of being married or cohabiting_._ Studying four cohorts with different disease duration can, although not truly longitudinal, still give information on how the duration of diabetes impacts HRQoL beyond the effects of ageing.

As the excess mortality associated with diabetes [1] is expected to decrease due to better diabetes management, measures like HRQoL increase in importance. Many of the national quality registers in Sweden contain patient-reported outcomes [37]. An increased use of patient-reported outcome measures could emphasise the patient’s perspective and increase the opportunities for patients to become more active in the management of their diabetes. This could facilitate further improvement of quality and management of healthcare services for people with diabetes and other chronic diseases.

## Conclusion

Our study confirms the overall negative impact of diabetes on HRQoL and that the differences between individuals with diabetes and control individuals tend to increase with longer disease duration. HRQoL was lower already one year after diagnosis although to a limited extent, which could imply a good management of diabetes care and quick adaptation by the newly diagnosed. Our results also suggest an earlier appearance and a stronger negative impact on HRQoL for women, which should be considered in further development of diabetes management and treatment in Sweden.

## Competing interests

The authors declare that they have no competing interests.

## Authors’ contributions

VS designed the study, analysed the data and wrote, reviewed and edited the manuscript, PMJ conceptualised and designed the study and reviewed the manuscript, LN, RW, JÖ contributed to the discussion and reviewed the manuscript, and KB interpreted the data, contributed to the discussion and reviewed and edited the manuscript. All authors read and approved the final manuscript.

## Pre-publication history

The pre-publication history for this paper can be accessed here:

http://www.biomedcentral.com/1471-2458/13/377/prepub
